# Surgical Applications of Lymphatic Vessel Visualization Using Photoacoustic Imaging and Augmented Reality

**DOI:** 10.3390/jcm11010194

**Published:** 2021-12-30

**Authors:** Yushi Suzuki, Hiroki Kajita, Shiho Watanabe, Marika Otaki, Keisuke Okabe, Hisashi Sakuma, Yoshifumi Takatsume, Nobuaki Imanishi, Sadakazu Aiso, Kazuo Kishi

**Affiliations:** 1 Department of Plastic and Reconstructive Surgery, Keio University School of Medicine, 35 Shinanomachi, Shinjuku-ku, Tokyo 160-8582, Japan; jmrbx767@keio.jp (H.K.); swatanabe.3210@gmail.com (S.W.); otakimarika@gmail.com (M.O.); dawndawn@keio.jp (K.O.); kkishi@a7.keio.jp (K.K.); 2 Department of Plastic and Reconstructive Surgery, Tokyo Dental College Ichikawa General Hospital, Chiba 272-8513, Japan; prssakuma@gmail.com; 3 Department of Anatomy, Keio University School of Medicine, Tokyo 160-8582, Japan; tsume@keio.jp (Y.T.); nimanmed@z5.keio.jp (N.I.); 4 Luxonus Inc., Kawasaki 212-0032, Kanagawa, Japan; aiso.sadakazu@luxonus.jp

**Keywords:** photoacoustic imaging, photoacoustic lymphangiography, lymphedema, lymphaticovenular anastomosis, augmented reality

## Abstract

Lymphaticovenular anastomosis (LVA) is a widely performed surgical procedure for the treatment of lymphedema. For good LVA outcomes, identifying lymphatic vessels and venules is crucial. Photoacoustic lymphangiography (PAL) is a new technology for visualizing lymphatic vessels. It can depict lymphatic vessels at high resolution; therefore, this study focused on how to apply PAL for lymphatic surgery. To visualize lymphatic vessels, indocyanine green was injected as a color agent. PAI-05 was used as the photoacoustic imaging device. Lymphatic vessels and veins were visualized at 797- and 835-nm wavelengths. First, it was confirmed whether the branching of the vasculature as depicted by the PAL was consistent with the actual branching of the vasculature as confirmed intraoperatively. Second, to use PAL images for surgical planning, preoperative photoacoustic images were superimposed onto the patient limb through augmented reality (AR) glasses (MOVERIO Smart Glass BT-30E). Lymphatics and venule markings drawn using AR glasses were consistent with the actual intraoperative images obtained during LVA. To anastomose multiple lymphatic vessels, a site with abundant venous branching was selected as the incision site; and selecting the incision site became easier. The anatomical morphology obtained by PAL matched the surgical field. AR-based marking could be very useful in future LVA.

## 1. Introduction

Lymphaticovenular anastomosis (LVA) is a widely performed surgical procedure for the treatment of lymphedema. The congested lymphatic flow due to lymphedema is bypassed into a vein to improve edema symptoms [1,2,3]. While LVA is less effective for advanced lymphedema patients, the surgical procedure is minimally invasive and has high efficacy for early to moderate stage lymphedema patients [4,5,6]. Since a good surgical outcome can be obtained by making as many anastomoses as possible between lymphatic vessels and veins [7], rapid identification of lymphatic vessels and veins is necessary for efficient surgery. Lymphoscintigraphy [8], near-infrared fluorescence lymphangiography (NIRF) [9,10], ultrasonography [11,12], and magnetic resonance lymphangiography [13] are well-known modalities for identifying lymphatic vessels. In addition, there is a recent method of identifying lymphatic vessels using a technique called photoacoustic imaging (PAI) [14,15]. Lymphatic vessels in patients with lymphedema deteriorate as edema gradually progresses, as shown in a study using NIRF [10]. Since the images obtained by PAI correlate with NIRF, this technique may be useful in establishing a preoperative plan for LVA patients [16]. Herein, this paper introduces a new imaging technique to facilitate the surgical treatment of lymphedema.

## 2. Materials and Methods

### 2.1. Principles of Photoacoustic Imaging

When an object is repeatedly irradiated with a laser beam, it absorbs the energy of the light and emits ultrasound waves generated by the contraction and expansion of itself. The principle of photoacoustic imaging is to create an image by detecting the ultrasonic waves.

**Figure 1:**
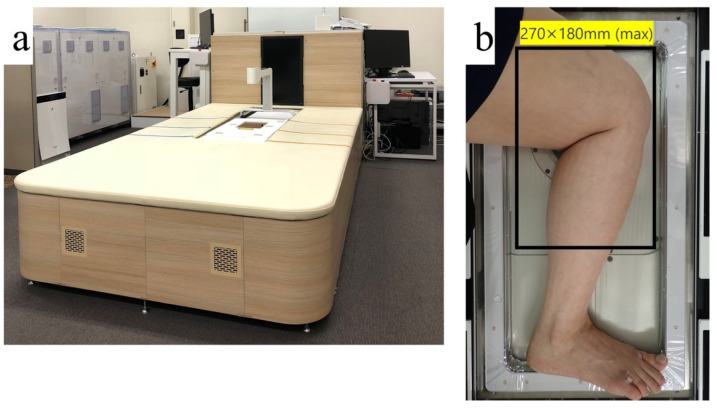
(**a**) Appearance of the imaging system PAI-05 (Luxonus, Kanagawa, Japan). (**b**) To take a picture of the inside of the knee, the knee is placed inside the frame, as shown here; then it is possible to take a picture in the range of 270 × 180 mm, as shown by the rectangle. Adapted in part from [15].

This study used PAI-05 (Luxonus, Kanagawa, Japan) as a PAI device. PAI-05 has a hemisphere-shaped sensor at the center of the large bed. PAI figures can be obtained by positioning the object to be imaged on the sensor (Figure 1). Because lymph fluid is colorless, it cannot delineate lymphatic vessels. Thus, indocyanine green (ICG; Diagnogreen 0.5%; Daiichi Pharmaceutical, Tokyo, Japan) was injected as a color agent to visualize the lymphatic vessels. Moreover, PAI-05 can irradiate two different wavelengths. To identify lymphatic vessels and venules simultaneously, 797- and 835-nm lasers were chosen to irradiate the object, which were appropriate for hemoglobin and indocyanine green [15].

Furthermore, the machine was also capable of depicting three-dimensional structures by using a bowl-shaped sensor to receive ultrasound waves emitted from the object at multiple points. This made it possible to see the lymphatic vessels and blood vessels, including which ran deeper. This study was conducted in accordance with the standards of the ethics committee at the Certified Review Board of Keio (approval number: jRCTs032180204). 

#### 2.1.1. Method: Photoacoustic Imaging Match with Surgical Findings

The imaging examination that uses PAI to depict lymphatic vessels is called photoacoustic lymphangiography (PAL). Lymphatic vessels and veins can be clearly identified compared with NIRF [15,16]. First, for the surgical application of PAL technology, it is necessary to ensure that the PAL matches the actual surgical field. Patients who underwent PA imaging before LVA surgery, were retrospectively evaluated as to whether the surgical fields and PAI were matched. Regarding image matching, an author (YS) postoperatively evaluated whether the vein branching obtained by PAI actually corresponded to the intraoperative images.

#### 2.1.2. Method: Transfer of Photoacoustic Image to the Actual Surgical Field

In the future, high-resolution imaging will be very useful for the preoperative mapping of LVA. Even if high-resolution imaging can be obtained, it is not used if it cannot be projected to the correct position on the patient’s limb. To overcome this problem, augmented reality (AR) was used for positioning.

**Figure 2:**
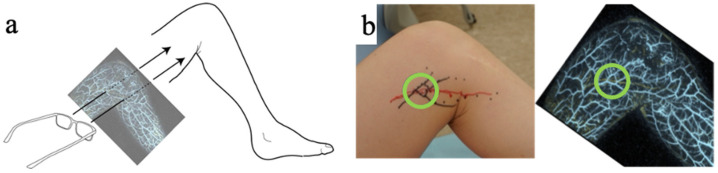
(**a**) Introduction of the marking technique, which allows marking of the affected limb by displaying the photoacoustic image taken on the AR glasses. (**b**) Marking of the area (circled) where two lymphatic vessels are running and there are many branches of veins to anastomose them together. Yellow lines indicate lymphatic vessels, while blue lines show the veins in photoacoustic lymphangiography.

Preoperative photoacoustic images were displayed on a PC, and these images were projected onto AR glasses (MOVERIO Smart Glass BT-30E, EPSON, Nagano, Japan). Since the lymphatic vessels and blood vessels were visible in the photoacoustic image, if they were properly aligned (i.e., their positions matched), the lymphatic vessels and blood vessels could be marked by tracing them (Figure 2, Video S1). Because MOVERIO could display the images onto the transparent lens, it was easy to superimpose the displayed image onto the patient. A surgeon aligned the outline of the lower limb on the photoacoustic image with the silhouette of the lower limb, to mark them. This study evaluated whether marking performed in this way had a positive effect on the surgery.

## 3. Results

### 3.1. Result: Photoacoustic Imaging Match with Surgical Findings

Five patients who underwent PAL and LVA at our hospital were included in the study. All five patients were female, with a mean age of 59.4 years, and had secondary lymphedema stage 2, according to the International Society of Lymphology (ISL) classification, after gynecologic cancer surgery.

Twelve incision sites were included in the study, and two sites were excluded because the obtained images could not be accurately superimposed on the actual surgical field. In the remaining 10 sites, consistency was checked between the obtained photoacoustic images and the intraoperative images.

**Figure 3:**
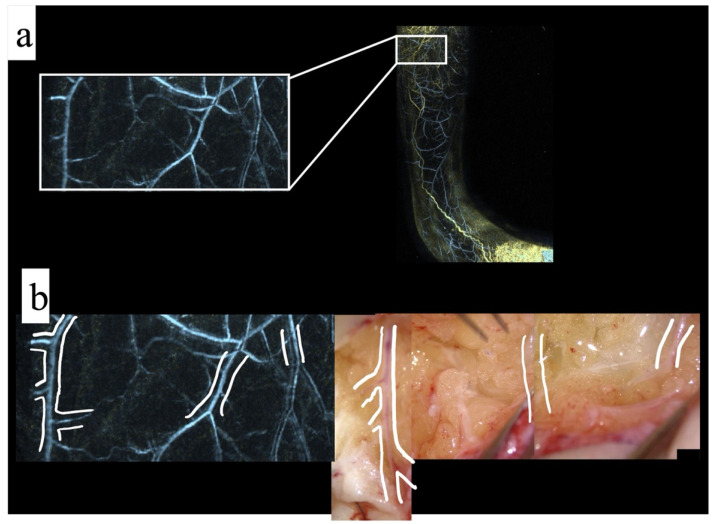
(**a**) Right: photoacoustic imaging of a 44 year old woman with secondary lymphedema on the lateral side of the lower leg, blue indicates veins; left: magnification of the area enclosed by the square. (**b**) Left: outline of the vein obtained in the magnified view; right: the venous branching pattern is almost identical to that obtained during surgery.

In reality, some of the tiny venous branches should be cauterized during surgery to approach the surgical field; thus, the results did not completely match to all branches. However, the thin branches depicted by PAI were consistent with the actual surgical field. The venous morphology obtained by PA imaging was consistent with the actual intraoperative images during LVA surgery (Figure 3).

Consequently, in all 10 sites, the obtained photoacoustic images were consistent with the images obtained from the intraoperative microscopic imaging. In other words, the PAI and the intraoperative images were consistent in all cases as long as they could be reliably superimposed.

### 3.2. Result: Transfer the Photoacoustic Image to the Actual Surgical Field

**Figure 4:**
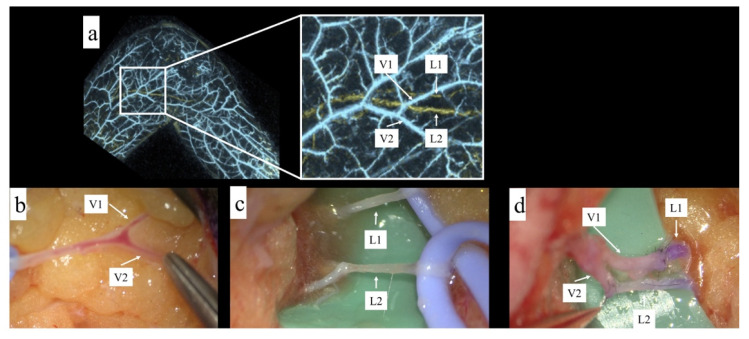
(**a**) The square was enlarged, and two lymphatic vessels and their superficial venous branches were identified. (**b**) The superficial veins and their branches were first identified. (**c**) Two lymphatic vessels were identified. (**d**) Two LVAs could be performed in one surgical field in a small incision.

A 58 year old woman with secondary lymphedema (ISL stage 2) after gynecologic cancer, who was scheduled for LVA surgery, was recruited for this trial. In this case, two lymphatic vessels had a straight pattern in PAL preoperatively. Therefore, by analyzing the photoacoustic image, the incision point was decided so that a Y-shaped venule could be identified for two LVA. Then, marking of the vessels was performed using MOVERIO. As a result, the two lymphatic vessels were quickly identified intraoperatively, and two lymphaticovenular end-to-end anastomoses were performed (Figure 4).

## 4. Discussion

In this study, the application of photoacoustic imaging was introduced for the surgical treatment of lymphedema. NIRF is a very useful method for the preoperative mapping of LVA because it can mark lymphatic vessels in real time. However, it is difficult to identify the partner vein for anastomosis when using NIRF. It is sometimes necessary to perform a vein graft or to make a larger incision when surgeons cannot find the anastomotic partner vein [17]. To prevent this scenario, ultrasonography can be used to identify blood vessels in advance, but the examination results vary depending on the examiner’s level of proficiency.

Conversely, since PAL can simultaneously depict lymphatic vessels and blood vessels, it is possible to quickly see how close the blood vessels run to the lymphatic vessels to be anastomosed. In addition, PAL can visualize high-resolution images, when compared with the previous modality. This makes it possible to identify the lymphatic vessels and veins more easily during the operation.

PAI has some room for improvement in terms of transferring the obtained image to the patient’s limb. In other words, incorrect superimposition and alignment render the vasculature image useless, regardless of its accuracy. This article introduced an approach using AR to transfer images. An approach that uses AR to transfer an image obtained by computed tomography or magnetic resonance imaging to the patient’s body has previously been used in the field of plastic surgery [18,19]. Accurate mapping is possible by referring to anatomical markers, such as the iliac bone and pubic bone. Furthermore, the AR device used in this study is a head-mounted display (HMD), which is also useful. The HMD allows for smooth marking as surgeons’ hands are free during marking compared with handheld devices [20].

Other methods for transferring the image obtained by photoacoustic imaging through a transparent film for accurate positioning [21] were also reported. In this study, it was easy to superimpose the displayed image on the silhouette of the lower limb by using the transparent HMD, MOVERIO. However, this method may cause misalignment during the transfer process depending on the body position and imaging location. The authors will conduct further investigations with more cases in the future, to identify better methods.

This study had limitations. As it was a preliminary study, the number of patients was small. Additionally, since many new multiple devices were used in this study, the evaluation of cost-effectiveness is also of concern. However, the PAI-05 used in this study is still a prototype machine undergoing clinical trials and is not yet available for sale. Therefore, it is difficult to evaluate its cost-effectiveness. Nevertheless, further studies should be performed to establish evidence on the usefulness of PAL. However, this high-resolution imaging technology is undoubtedly useful for lymphatic surgery.

## 5. Conclusions

The anatomical morphology obtained by PAL matched the surgical fields, and the AR-based marking could be very useful in future LVA.

## Supplementary Materials

The following are available online at https://www.mdpi.com/article/10.3390/jcm11010194/s1, Video S1: This video shows how photoacoustic imaging can be applied to preoperative mapping. The PC screen is displayed on the AR device, and marking is performed. AR: augmented reality.
